# Live bacteria found in gastric cancer tumor tissue

**DOI:** 10.3389/fmicb.2025.1591735

**Published:** 2025-06-18

**Authors:** Xiaoqian Lv, Pengxia Song, Nianhua Zhang, Pengbo Guo, Rujian Zhang, Haitao Wang, Longyan Hong, Rui Xu, Yu Wang, Gang Lv, Yinghui Zhao

**Affiliations:** ^1^Department of Clinical Laboratory Medicine, The first Affiliated Hospital of Shandong First Medical University & Shandong Provincial Qianfoshan Hospital, Laboratory of Metabolism and Gastrointestinal Tumor, Shandong, China; ^2^Department of Pathogenic Biology, School of Clinical and Basic Medicine, Shandong First Medical University, Jinan, Shandong, China; ^3^Medical School, Quzhou College of Technology, Quzhou, Zhejiang, China; ^4^Academic Registry, Shandong First Medical University, Jinan, Shandong, China; ^5^The Second Affiliated Hospital of Shandong First Medical University, Tai’an, Shandong, China

**Keywords:** gastric cancer, tumor tissue, Bacteria, *Propionibacterium acnes*, POF1B

## Abstract

**Background:**

Most research on gastric cancer and microorganisms has focused on *Helicobacter pylori* as a causative agent and on the relationship between the microbiota and gastric cancer. There is no direct evidence of bacteria in gastric cancer tissues or their relationship with gastric cancer.

**Methods:**

Gastric cancer tissue samples were collected, and the bacteria within tumor tissues were observed by transmission electron microscopy. The bacteria cultured were stained by Gram Staining and then subjected to High-throughput third-generation full-length 16S rRNA amplicon sequencing. Pure cultures were used to infect AGS gastric cancer cells, followed by protein extraction and Western blotting to detect POF1B protein expression.

**Results:**

The bacteria were observed in tumor tissues through transmission electron microscopy and had been cultivated under microaerophilic and anaerobic conditions, specifically, then were identified as *Propionibacterium acnes* (*P. acnes*), *Klebsiella pneumoniae (K. pneumoniae)*, and *Lactobacillus salivarius (L. salivarius)*. The vacuolation and mortality of AGS cell infected with *P. acnes* increased (*p* < 0.05) and the POF1B protein expression upregulated (*p* < 0.05).

**Conclusion:**

The live bacteria, including *L. salivarius*, *P. acnes*, and *K. pneumoniae*, have been found in gastric cancer tumor tissue. *P. acnes* specifically upregulate the oncogenic protein POF1B expression. The pathway through which this bacterium enters tumor tissue and its role in the tumor microenvironment require further investigation.

## Introduction

Cancer is one of the major diseases that threatens human health. Although significant progress has been made in cancer prevention and treatment, the mechanisms underlying tumorigenesis remain unclear. In recent years, intratumoral microbiota has become a research hotspot (Wong-Rolle et al., 2021). Significant differences have been observed in the microbial community structures between hepatocellular carcinoma tissues and normal tissues (Huang et al., 2022). Different types of tumors are characterized by distinct microbiota compositions (Geller et al., 2017). For instance, microbial communities have been identified in nasopharyngeal carcinoma (NPC) tissues, primarily consisting of *Corynebacterium* and *Staphylococcus* (Qiao et al., 2022). In breast cancer tissues, the abundance of *Lactobacillus* spp. and *Enterococcus* spp. is significantly higher, while normal breast tissues are dominated by *Staphylococcus* spp. and *Streptococcus* spp. (Urbaniak et al., 2014; Urbaniak et al., 2016; Hieken et al., 2016).

Recent studies have identified bacteria such as *Fusobacterium nucleatum* and *Porphyromonas gingivalis* in tumors, which are believed to promote tumorigenesis (Ou et al., 2022; Parhi et al., 2020; Stasiewicz and Karpiński, 2022). These bacteria assist tumors in evading immune surveillance, protect tumor cells from immune system attacks, and promote distant metastasis (Fu et al., 2022; Ferrari and Rescigno, 2023; Galeano Niño et al., 2022; Wang et al., 2023). However, it is still unclear whether there are microbial differences between gastric cancer tissues and normal tissues, and their role is worth studying.

Gastric cancer (GC) ranks 5th in worldwide cancer incidence, yet its mortality rate rises to 3rd. Early diagnosis is challenging, and patients are often diagnosed at advanced stages. It has a high recurrence rate, a median survival of less than a year, and a 5-year survival rate of under 50% (Smyth et al., 2020). Most research on gastric cancer and microorganisms has focused on *Helicobacter pylori (H. pylori)* as a causative agent and on the relationship between the microbiota and gastric cancer (Zeng et al., 2024; Zhu et al., 2022). In recent years, there have been reports of finding indirect evidence of bacteria in gastric cancer. 16S rRNA gene analysis showed those who had the highest level (highest tertile) of relative *H. pylori* and *P. acnes* abundances showed a significantly higher risk for GC (Gunathilake et al., 2019). Immunohistochemical staining using *P. acnes*-specific antibodies showed a large number of *P. acnes*-positive cells in the granulomas from early-stage gastric cancer (Tanaka et al., 2021). *P. acnes* significantly increased in GC tissues, especially in *H. pylori*-negative tissues by 16S rRNA gene sequencing and fluorescence *in situ* hybridization (FISH) (Li Q. et al., 2021). However, there is no evidence of live bacteria in gastric cancer tissue. This study aims to explore the presence and types of bacteria in gastric cancer tissues, laying a foundation for understanding the function and potential applications of intratumoral bacteria in gastric cancer.

## Materials and methods

### Specimens and cell line

Gastric cancer and adjacent non-cancerous tissue were collected from the Department of Gastrointestinal Surgery at the Second Affiliated Hospital of Shandong First Medical University, with informed consent from patients and approval by the Shandong first medical university’s Ethics Committee (2023-854). The cancer type was determined as mucinous adenocarcinoma stage I. The gastric cancer cell line AGS was purchased from Procell Biotechnology Co., Ltd. (Wuhan, China).

### Key reagents and instruments

Fixative solution for electron microscopy and Gram staining reagents were obtained from Solarbio (P1127-100ml, G1060, China). Columbia blood agar plates were obtained from Hopebio (HBPM0153, China). Sterile DMEM medium was purchased from Gibco (11965092, United States). NucleoBond^®^ HMW DNA kit was acquired from MN (740160.20, Germany). RIPA lysis buffer/BCA protein assay kit was obtained from Solarbio (R0010/PC0020, China). PVDF membranes were purchased from Millipore (IPVH00010, USA). POF1B primary antibody and HRP-conjugated secondary antibody were obtained from Proteintech (11398-1-AP/SA00001-2, China). ECL chemiluminescence substrate kit (ultrasensitive) was purchased from Bioground (BL523B, China).

Fluorescence quantification instrument Qubit4.0 (Thermo, Q33226, United States); Nanodrop micro-spectrophotometer (SMA4000, Taiwan, China); Agencourt AMPure XP Beads (Beckman, A63881, USA); MinION Flow Cell for nanopore sequencing (ONT, R9.4.1, United Kingdom); nextpolish correction software (v1.4.1, China); Pilon genome correction software (v1.18, United States).

Transmission electron microscope HT7800/HT7700 (HITACHI, Japan); incubator IMP180 (Thermo, United States); anaerobic system ANOXOMAT SMA4000 (MART, Netherlands); MARKIII cultivation system (Wiggens, Germany); high-throughput sequencing instrument Illumina NovaSeq 6000 (Illumina, United States).

### Sample collection and identification

Three samples of gastric cancer and adjacent non-cancerous tissues were collected under sterile conditions. The part of each fresh tissue sample was sent to the pathology department, where it was fixed, sectioned, and stained with H&E. Another portion was promptly transferred to a 1.5 ml conical tube containing sterile DMEM for electron microscopy observation and bacterial culture.

### Transmission electron microscopy observation of intratumoral bacteria

One mm^3^ tissue block was fixed for 2 hours, washed with sterile pre-chilled PBS, and re-fixed. The tissue was washed again with sterile pre-chilled PBS, dehydrated, embedded, and sectioned into 70 nm slices. The sections were double-stained with 3% uranyl acetate and lead citrate for transmission electron microscopy (TEM) observation to obtain high-resolution images of bacteria within the tissue.

### Isolation and culture of bacteria from gastric cancer tissue

Under sterile conditions, the external surface of the gastric cancer and adjacent tissue was disinfected. Approximately 0.05 g of internal tumor tissue was cut, minced, and added to 1 mL of sterile pre-chilled PBS. The suspension was diluted tenfold with PBS and plated onto standard agar plates, Columbia blood agar plates, and egg yolk plates. Plates were incubated in various gas environments (anaerobic, 5% O_2_, aerobic, and 5% O_2_, 10% CO2, 85% N_2_) at 37°C for 72 h to isolate single colonies, which were then purified.

### Gram staining

The sterile glass slide was prepared with 10 μL of saline. A bacterial colony was picked with an inoculation loop and spread onto the slide to form a thin layer. After air drying, the sample was heat-fixed above a flame. The slide was stained with 100 μL of 1% crystal violet solution for 1 min, washed, and dried. Next, 100 μL of 0.5% iodine solution was applied for 1 min, followed by washing and drying. Decolorization was done with 100 μL of 95% ethanol for 1 min, then washed and dried. Finally, 100 μL of 0.5% safranin was applied for 1 min. The slide was washed, dried, and observed under a microscope.

### High-throughput third-generation 16S full-length sequencing

Genomic DNA was extracted from tissue samples using the NucleoBond^®^ HMW DNA kit, and DNA concentration and purity were measured with Qubit 4.0 and Nanodrop. DNA integrity was evaluated by 0.75% agarose gel electrophoresis. Universal primers 27F-1492R were used to amplify the full-length 16S rRNA gene, and the PCR product was purified with Agencourt AMPure XP Beads. The sequencing library was constructed with specific adapters and sequenced on a PacBio platform, generating approximately 10,000 high-quality sequences per sample. Data were filtered and assembled using Canu (default parameters), and genome sequences were corrected with nextpolish (v1.4.1) and Pilon (v1.18). Taxonomic identification was performed using the SILVA database,^1^ and average nucleotide identity (ANI) between samples was calculated using the ANI calculator.^2^ Comparative genomic analysis was conducted using the Bacterial Pan Genome Analysis (BPGA) pipeline to explore genomic differences between samples. Results were visualized in charts and graphs for interpretation.

### Analysis of POF1B gene expression in gastric cancer tissues

The Gene Expression Profiling Interactive Analysis (GEPIA)^3^ online tool was used to compare the expression of the POF1B gene between gastric cancer and normal tissues based on standardized RNA-seq data from the TCGA and GTEx databases.

### Cell morphology experiment

The AGS cells were cultured to the logarithmic phase and infected with *P. acnes*, *L. salivarius*, and *K. pneumoniae* (MOI = 1). The cell morphology was observed at 0, 4, 8, and 12 h after infected.

### Western blot

At 8 h post-infection, cell samples were collected, and protein was extracted and quantified using the BCA. Protein samples (10 μL, 2 μg/μL) were separated by electrophoresis, transferred to membranes, and blocked with 5% nonfat milk for 1 h. Membranes were incubated overnight at 4°C with POF1B primary antibody (1:2,000). After washing with TBST three times (5 min each), membranes were incubated at room temperature for 1 h with HRP-conjugated secondary antibody (1:5,000). Visualization was performed using ECL substrate, and bands were imaged using a gel imaging system. POF1B protein expression were quantified by analyzing grayscale values using ImageJ.

### Statistical analysis

All experiments were performed in triplicate. Means and standard deviations were calculated using GraphPad Prism 8.0, and statistical significance was analyzed using SPSS22.0, with *t*-tests and other statistical methods.

## Results

### Observation of microorganisms in tumor tissue via transmission electron microscopy

**FIGURE 1:**
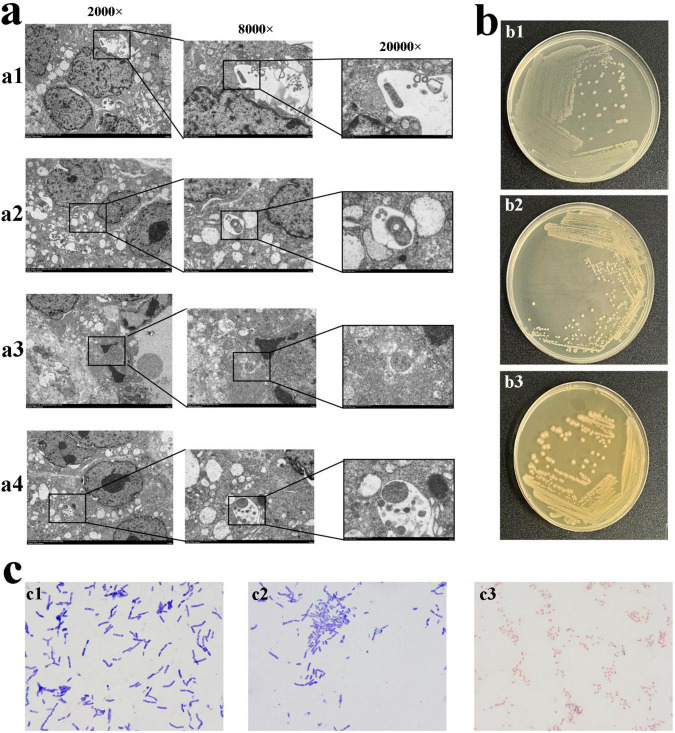
The morphology of bacteria in tumor tissue. **(a)** TEM observation of bacteria in tumor tissue. The bacteria in gastric cancer tissue from sample 1 **(a1)**. The bacteria observed in gastric cancer tissue from sample 2 **(a2,a3)**. The bacteria observed in gastric cancer tissue from sample 3 **(a4)**. **(b)** The bacteria obtained after 72 h of cultivation in high-nutrient anaerobic environment. **(b1)** Colony b1, small, white, approximately 1.5∼2 mm in diameter, circular with regular edges, smooth, moist surface, creamy texture, slightly elevated, opaque, cultured in a low-nutrient and no O_2_. **(b2)** Colony b2, white, 0.5∼1.5 mm in diameter, circular with regular edges, smooth, moist surface, opaque, cultured in a high-nutrient and 5% O_2_. **(b3)** Colony b3, Grayish-white, 1.5∼2.5 mm in diameter, circular with smooth, glossy edges, sticky texture, cultured in a high-nutrient and 5% O_2_. **(c)** Gram staining of pure cultures of colonies b1, b2, and b3 (1,000 × Oil Immersion). **(c1)** Colony b1, blue-violet, rod-shaped bacteria with one blunt, rounded end and one tapered end, arranged singly, in pairs, or in short chains. **(c2)** Colony b2, blue-violet, slender rod-shaped bacteria with blunt-rounded ends, arranged singly or in chains. **(c3)** Colony b3, red, short and thick rod-shaped bacteria with blunt-rounded ends, arranged singly, in pairs, or in chains.

To directly observe bacteria in gastric cancer tissue, TEM imaging was performed on gastric cancer and adjacent non-cancerous tissue samples. Tumor tissue was examined at magnifications of 2,000 × (Figure 1a), 8,000 × (Figure 1a), and 20,000 × (Figure 1a). Rod-shaped bacteria were observed within the tumor tissue of three samples, and coccoid bacteria were observed within the tumor tissue of sample 2, 3, whereas no bacteria were detected in the adjacent non-cancerous tissue.

### Pure cultivation of bacteria in tumor tissue

To investigate whether bacteria are alive in tumors, after 72 h of cultivation, the bacterial colonies were observed. In a high-nutrient, anaerobic environment, colony b1 appeared small, white, and circular with a diameter of approximately 1.5∼2 mm. It had regular edges, a smooth and moist surface, and a creamy texture with a slight elevation, appearing opaque (Figures 1b,b1). Under high-nutrient conditions with 5% O_2_, colony b2 appeared diameter of 0.5∼1.5 mm, white, circular, with regular edges and a smooth, moist, opaque surface (Figures 1b,b2). In the same 5% O_2_ environment, colony b3 appeared grayish-white, approximately 1.5∼2.5 mm in diameter, circular with smooth and glossy edges, and had a sticky texture (Figures 1b,b3). Colony b1, b2 were cultured from three samples, and Colony b3 was cultured from sample 2, 3. There was no bacteria in the adjacent non-cancerous tissue, and *H. pylori*, as a key biological factor in gastric cancer, was not detected in the cultures.

### Gram staining microscopy

To determine the staining characteristics and morphology, the cultured bacteria were subjected to Gram staining, the bacteria of colony b1 appeared blue-violet, with a rod shape, one end blunt and rounded, the other end tapered. They were observed singly, in pairs, or short chains (Figures 1c,c1). The bacteria of colony b2 appeared blue-violet, with a slender rod shape and blunt-rounded ends. They were observed singly or in chains (Figures 1c,c2). The bacteria of colony b3 appeared red, with a short and thick rod shape and blunt-rounded ends. They were observed singly, in pairs, or in chains (Figures 1c,c3).

### Bacterial identification in tumor tissue

**FIGURE 2:**
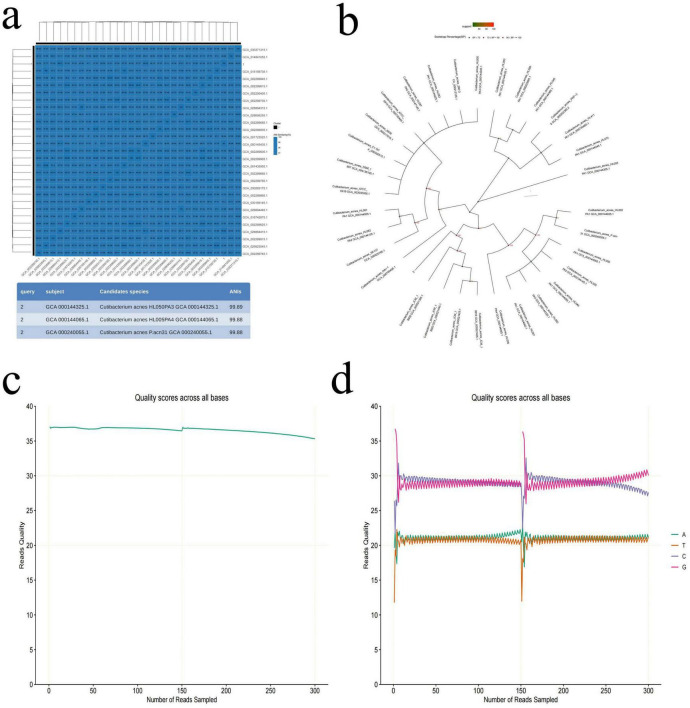
*P. acne* high-throughput third-generation 16S full-length sequencing. **(a)** Average nucleotide identity (ANI) similarity heatmap for *P. acnes*. This heatmap visually displays the ANI values between the sample numbers and the reference sequence accession numbers, where darker colors indicate higher ANI, suggesting closer similarity between strains. Strains with an ANI greater than 95% are considered to belong to the same species. **(b)** Phylogenetic tree of *P. acnes* based on the 16S rRNA gene sequence, showing the evolutionary relationships of *P. acnes* with other strains. **(c)** Quality control curve for *P. acnes*, providing data on the sequencing quality to ensure reliability. **(d)** Sequencing of *P. acnes*, displaying sequence characteristics that support the classification analysis results.

**FIGURE 3:**
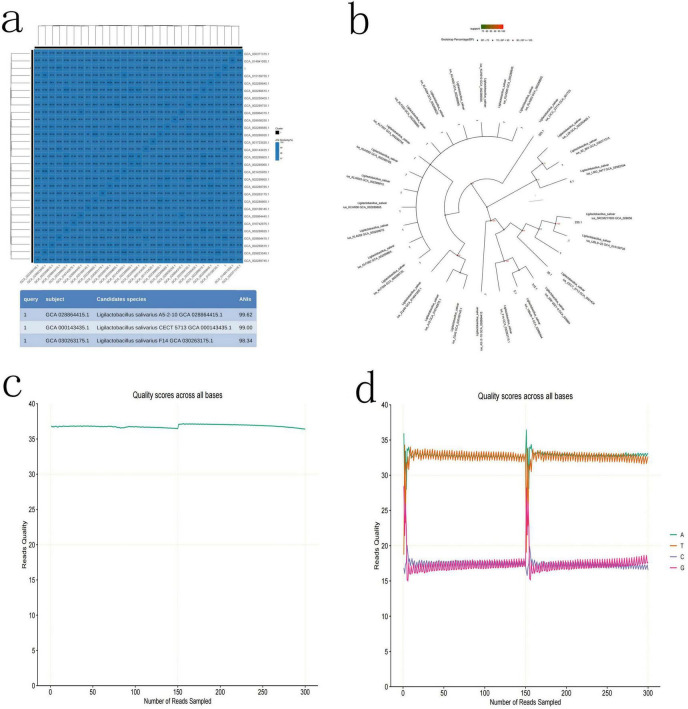
*L. salivarius* high-throughput third-generation 16S full-length sequencing. **(a)** Average Nucleotide Identity (ANI) Similarity Heatmap for *L. salivarius*, with color depth indicating the similarity between samples. **(b)** Phylogenetic tree of *L. salivarius* based on the 16S rRNA gene sequence, illustrating the evolutionary relationships with other strains. **(c)** Quality control curve for *L. salivarius*, ensuring the reliability of the sequencing results. **(d)** Sequencing of *L. salivarius*, highlighting the sequence characteristics of the strain.

**FIGURE 4:**
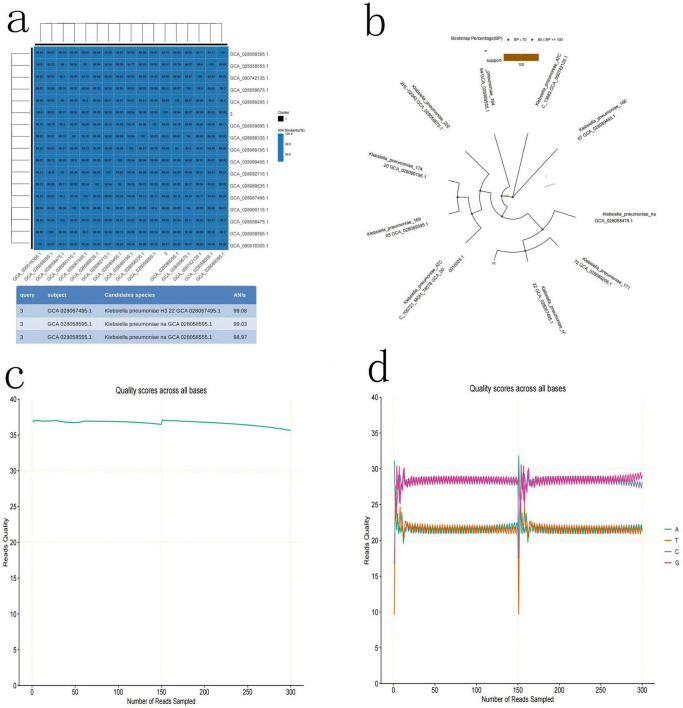
*K. pneumoniae* high-throughput third-generation 16S full-length sequencing. **(a)** Average nucleotide identity (ANI) Similarity Heatmap for *K. pneumoniae*. **(b)** Phylogenetic tree of *K. pneumoniae* based on the 16S rRNA gene sequence, showing its evolutionary relations. **(c)** Quality control curve for *K. pneumoniae*. **(d)** Sequencing results of *K. pneumoniae*, demonstrating the sequence features of the strain.

To determine which types of bacteria they are, high-throughput third-generation 16S full-length sequencing was used for bacterial species identification. The results revealed that Colony b1 was identified as *P. acnes* (Figures 2a–d); Colony b2 was identified as *L. salivarius* (Figures 3a–d); Colony b3 was identified as *K. pneumoniae* (Figures 4a–d).

### Cell morphology changes in after bacterial infection

**FIGURE 5:**
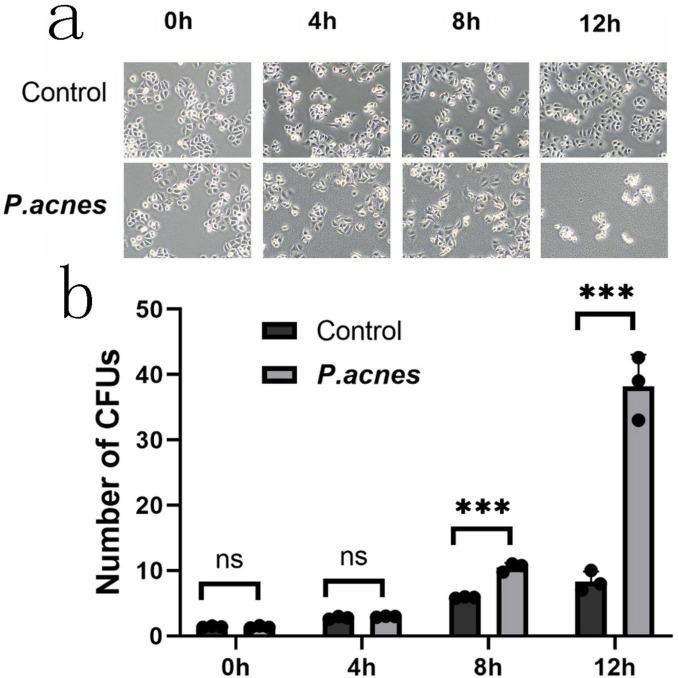
Morphological changes in AGS cells infected with *P. acnes.* **(a)** Morphological changes in AGS cells at 0, 4, 8, and 12 h post-*P. acnes* infection. **(b)** Quantification of the relative total area of cell vacuoles. ***p < 0.001.

To determine the impact of the bacteria on gastric cancer cells, AGS cells were infected with *P. acnes*, *L. salivarius*, and *K. pneumoniae* at a multiplicity of infection (MOI) of 1. Morphology was observed at 0, 4, 8, and 12 h post-infection. The results revealed that as the infection time increased, compared to the control group, the *P. acnes* infection group exhibited noticeable morphological abnormalities, such as an increase in cell vacuolation *(p* < 0.05) and an elevated cell death rate (*p* < 0.05) (Figures 5a,b).

### POF1B gene expression by bioinformatics analysis

**FIGURE 6:**
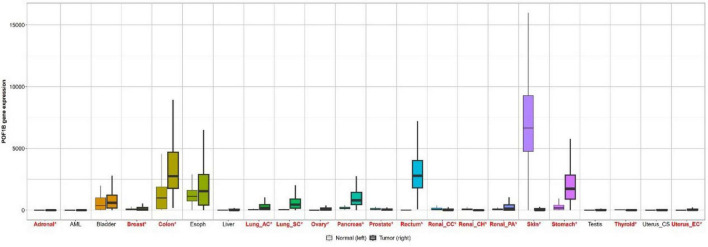
Bar chart comparing POF1B gene expression in normal tissues and cancer tissues based on data from the GEPIA website.

Based on the analysis of TCGA and GTEx databases using the GEPIA, it was found that the expression of the POF1B gene is significantly higher in gastric cancer tissues (Figure 6).

### Biological function of POF1B protein expression

**FIGURE 7:**
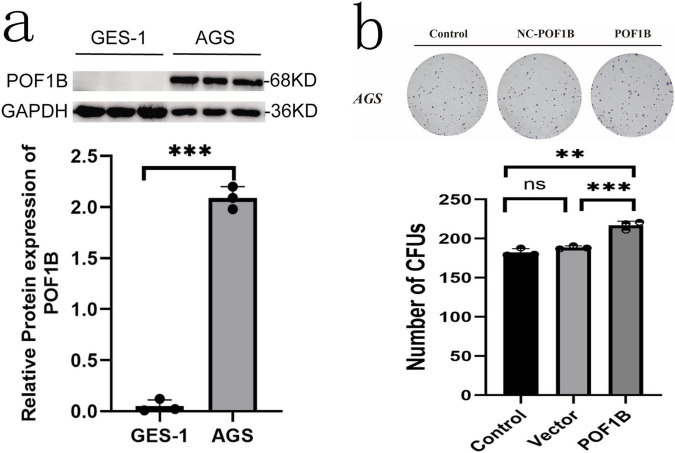
POF1B expression cell biology effects. **(a)** POF1B is expressed higher in AGS (poorly differentiated gastric cancer cell) cells than that in GES-1 (well differentiated cell) cells. POF1B expression statistical analysis. **(b)** Increased expression of POF1B enhances AGS cell proliferation ability. AGS cell proliferation ability statistical analysis. **p < 0.01, ***p < 0.001.

Our preliminary experiments have found that POF1B protein expression was higher in AGS (poorly differentiated gastric adenocarcinoma epithelial cell) cells than that in GES-1 (normal epithelial immortalized cell) cells (Figure 7a). After stable expression of POB1B vector was transfected into AGS cells, the cell colony forming ability and migration were enhanced (Figure 7b).

### POB1B protein changes of AGS cells Infected with bacteria

**FIGURE 8:**
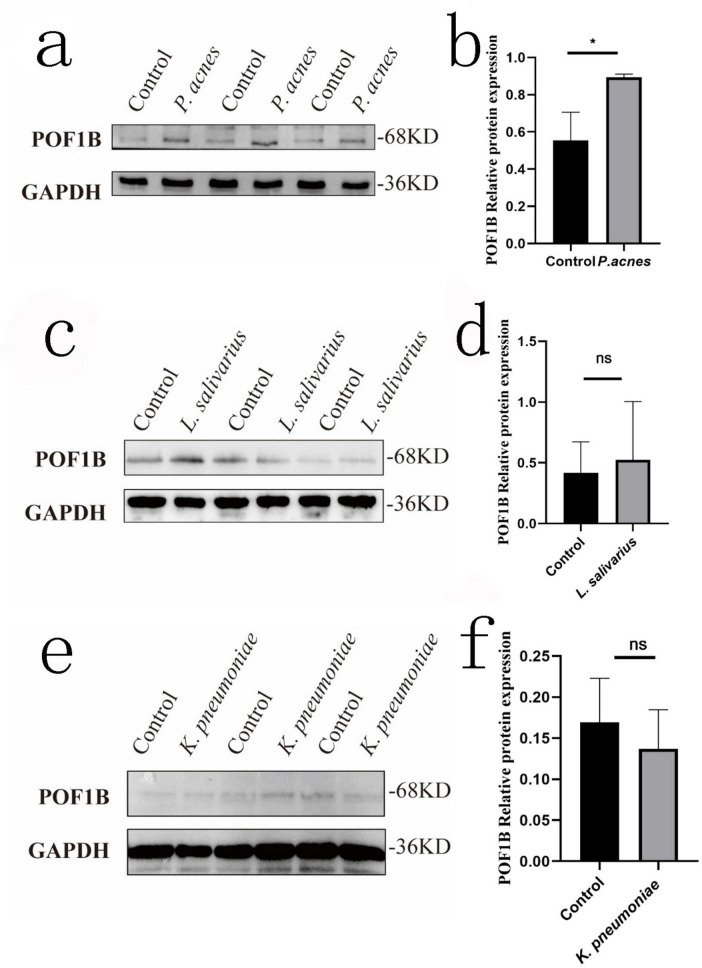
POF1B expression of AGS cells after infected *with P. acnes.* The figure shows the effect of *P. acnes*, *L. salivarius*, and *K. pneumoniae* on POF1B protein expression in AGS cells. The cells were infected with these three bacteria at a multiplicity of infection (MOI) = 1, and the proteins were extracted after infected 8 h for Western Blot of POF1B expression. **(a)** POF1B expression in AGS cells after *P. acnes* infection. **(b)** Quantification of relative gray values after *P. acnes* infection. **(c)** POF1B expression in AGS cells after *L. salivarius* infection. **(d)** Quantification of relative gray values after *L. salivarius* infection. **(e)** POF1B expression in AGS cells after *K. pneumoniae* infection. **(f)** Quantification of relative gray values after *K. pneumoniae* infection. *p < 0.05. ns, *p* > 0.05.

Based on the above results about POF1B, after AGS cells infected with *P. acnes*, *L. salivarius*, and *K. pneumoniae* for 8 h, the cell protein was extracted for Western blot to investigate the expression of POF1B. The results showed that *P. acnes* led to a significant increase in the expression of POF1B protein compared to the control group (*p* < 0.05) (Figures 8a,b). But, *L. salivarius* and *K. pneumoniae* didn’t result in a significant increase of the protein (Figures 8c–f) (*p* > 0.05).

## Discussion

In recent years, the microorganisms within various tumor tissues attracted the attention of many researchers. This study focuses on the microorganisms in the tumor tissue of gastric cancer, a common malignancy in humans. Although *H. pylori* is considered the primary biological factor responsible for gastric cancer, our study did not detect the bacterium in gastric cancer tissue, which aligns with previous research. Most studies suggest that *H. pylori* primarily colonizes the gastric mucosal epithelium, causing gastric cancer cell transformation, leading to gastric cancer or mucosa-associated lymphoid tissue lymphoma (Wang et al., 2024; He et al., 2024; Pan et al., 2024), etc.

Through transmission electron microscopy, the presence of bacilli and cocci in gastric cancer tissue were observed, but the cocci did not isolated and cultured *in vitro*, suggesting that they might be non-viable bacteria. What intrigued us was the successful isolation and identification of bacilli, including *P. acnes*, *K. pneumoniae*, and *L. salivarius*, all of which are extracellular bacteria.

*P. acnes* is a common facultative anaerobic Gram-positive bacterium found on the skin, particularly in hair follicles and sebaceous glands. When sebaceous glands are overactive, the bacterium breaks down triglycerides into free fatty acids, stimulating keratinization of the follicular duct and leading to blocked pores, acne, and pimples. Previous studies have found indirectly the presence of *P. acne* 16S rRNA and antigens in gastric cancer tissue (Gunathilake et al., 2019; Tanaka et al., 2021; Li Q. et al., 2021). Our results showed that *P. acnes* were found in GC tissues by electron microscope, furthermore, it was identified by isolating culture, staining and sequencing. Its role in gastric cancer has drawn attention. The abundance of *P. acnes* significantly increases in gastric cancer tissues and is associated with the TNM (tumor node metastasis) stage of gastric cancer (Li Q. et al., 2021). Encouragingly, its role and mechanism in gastric cancer need further investigation.

*K. pneumoniae* is a Gram-negative facultative anaerobic bacterium primarily found in the upper respiratory and intestinal tracts. When the immune system is compromised, it can enter the lungs via the respiratory tract and cause pneumonia. Although *K. pneumoniae* is not a primary gastric pathogen, microbiome analysis has suggested that it may play a role in the development of colorectal cancer, possibly influencing gastric cancer through similar mechanisms (Strakova et al., 2021). Excitingly, this bacterium in gastric cancer tissue was found firstly, and its mechanisms need further exploration.

*L. salivarius*, a Gram-positive facultative anaerobic bacterium belonging to the *Lactobacillus genus*, is primarily found in the human oral cavity. It plays an essential role in oral health by metabolizing organic compounds in the mouth, reducing the production of volatile organic compounds, and improving bad breath. The relative abundance of *L. salivarius* increases in gastric cancer patients, so its role in cancer progression may not always be beneficial. In a healthy stomach environment, the acidic conditions usually inhibit the growth of most bacteria. However, under pathological conditions such as chronic atrophic gastritis or gastric cancer, changes in stomach acidity may promote the overgrowth of microbes like *Lactobacillus genus*. This overgrowth may be associated with gastric mucosal inflammation and damage, potentially promoting gastric cancer development (Li Z. P. et al., 2021). *L. salivarius* have been isolated from the blood of patients with liver metastasis from gastric cancer (Lee et al., 2015). This is the first time *L. salivarius* has been found in gastric cancer tissue, and its roles need further exploration.

This study investigates the role of *P. acnes*, *K. pneumoniae*, and *L. salivarius* in gastric cancer tissue. Li et al report that *P. acnes* promote M2 polarization of macrophages through the TLR4/PI3K/Akt pathway, thereby promoting gastric cancer progression (Li Q. et al., 2021). It is worth studying whether this bacterium promotes the progression of gastric cancer in other ways.

POF1B is expressed in epithelial cells (Padovano et al., 2011; Bione et al., 2004). It interacts with non-muscle actin filaments and is localized to adherens junctions, regulating cell adhesion in human intestinal and keratinocyte cell (Lacombe et al., 2006; Crespi et al., 2015). Recent studies have found that abnormal POF1B expression plays a significant role in the progression, invasion, and prognosis of squamous cell carcinoma, mucinous ovarian tumors, colorectal cancer, bladder cancer, and lung adenocarcinoma (Bech et al., 2023; Zhang et al., 2023; Wang et al., 2021; Furtado et al., 2024; Ojeda et al., 2011).

Our bioinformatics analysis of clinical specimens showed an increase in POF1B gene expression. Its protein expression was higher in poorly differentiated gastric cancer cells than in well differentiated gastric cancer cells. When the protein expression increases, the colony forming ability of cells also increases.

So, after gastric cancer cells were infected with these bacteria, the POF1B expression were measured. Unfortunately, the changes in POF1B expression due to *K. pneumoniae* and *L. salivarius* infections were not observed, and their role in gastric cancer progression needs further confirmation. However, the results suggested that *P. acnes* led to increased POF1B expression in gastric cancer cells, whether this may be one of the mechanisms by which this bacterium promotes gastric cancer progression needs further validation in animal models and clinical cases. This finding provides new insights into the interaction between the gastric cancer microbiota and tumor gene expression and lays the foundation for exploring POF1B as a potential biomarker or therapeutic target for early diagnosis and intervention of gastric cancer.

However, the exact source of these bacteria in tumor tissue and their specific role in the tumor microenvironment remain largely unknown. Future research should further investigate the key questions, such as, how do the bacteria enter and colonize gastric cancer tissue? What are the potential pathways and mechanisms of invasion? What role do these bacteria play in the onset and progression of gastric cancer? What interactions exist between these bacteria and tumor cells? Does the presence of these bacteria affect gastric cancer diagnosis, treatment response, and prognosis? Can new prevention and treatment strategies be developed targeting these bacteria?

By addressing these questions, we may unravel the complexity of the gastric cancer microbiome, provide deeper insights into the mechanisms underlying gastric cancer development, and offer new theoretical and practical guidance for gastric cancer prevention, precision diagnosis, and individualized treatment. This will not only advance the field of gastric cancer research but also provide valuable experience and inspiration for microbiome studies in other cancers.

## Conclusion

In conclusion, this study identifies the presence of bacilli in gastric cancer tissue, including *P. acnes*, *K. pneumoniae*, and *L. salivarius*. Among these, *P. acnes* were found to increase POF1B expression of the cancer-related protein. The pathway through which this bacterium enters tumor tissue and its role in the tumor microenvironment require further investigation.

## Data Availability

The original contributions presented in the study are publicly available. This data can be found here: https://www.ncbi.nlm.nih.gov/, accession number: PRJNA1272491.

## Footnotes

1
www.arb-silva.de

2
www.ezbiocloud.net/tools/ANI

3
http://gepia.cancer-pku.cn/detail.php?gene=SEMA6D&clicktag=expdiy

## References

[B1] Bech J. M. Terkelsen T. Bartels A. S. Coscia F. Doll S. Zhao S. (2023). Proteomic profiling of colorectal adenomas identifies a predictive risk signature for development of metachronous advanced colorectal neoplasia. *Gastroenterology* 165 121–132.e5. 10.1053/j.gastro.2023.03.208 36966943

[B2] Bione S. Rizzolio F. Sala C. Ricotti R. Goegan M. Manzini M. C. (2004). Mutation analysis of two candidate genes for premature ovarian failure, DACH2 and POF1B. *Hum. Reprod.* 19 2759–2766. 10.1093/humrep/deh502 15459172

[B3] Crespi A. Bertoni A. Ferrari I. Padovano V. Della Mina P. Berti E. (2015). POF1B localizes to desmosomes and regulates cell adhesion in human intestinal and keratinocyte cell lines. *J. Invest. Dermatol.* 135 192–201. 10.1038/jid.2014.327 25084053

[B4] Ferrari V. Rescigno M. (2023). The intratumoral microbiota: Friend or foe? *Trends Cancer* 9 472–479. 10.1016/j.trecan.2023.03.005 37061408

[B5] Fu A. Yao B. Dong T. Chen Y. Yao J. Liu Y. (2022). Tumor-resident intracellular microbiota promotes metastatic colonization in breast cancer. *Cell* 185 1356–1372.e26. 10.1016/j.cell.2022.02.027 35395179

[B6] Furtado C. L. M. Soares M. R. Verruma C. G. de Oliveira Gennaro F. G. da Silva L. E. C. M. Ferriani R. A. (2024). BCORL1, POF1B, and USP9X copy number variation in women with idiopathic diminished ovarian reserve. *J. Assist. Reprod. Genet.* 41 2279–2288. 10.1007/s10815-024-03185-8 38995507 PMC11405560

[B7] Galeano Niño J. L. Wu H. LaCourse K. D. Kempchinsky A. G. Baryiames A. Barber B. (2022). Effect of the intratumoral microbiota on spatial and cellular heterogeneity in cancer. *Nature* 611 810–817. 10.1038/s41586-022-05435-0 36385528 PMC9684076

[B8] Geller L. T. Barzily-Rokni M. Danino T. Jonas O. H. Shental N. Nejman D. (2017). Potential role of intratumor bacteria in mediating tumor resistance to the chemotherapeutic drug gemcitabine. *Science* 357 1156–1160. 10.1126/science.aah5043 28912244 PMC5727343

[B9] Gunathilake M. Lee J. Choi I. Kim Y. Ahn Y. Park C. (2019). Association between the relative abundance of gastric microbiota and the risk of gastric cancer: A case-control study. *Sci. Rep.* 9:13589. 10.1038/s41598-019-50054-x 31537876 PMC6753194

[B10] He Y. Zhang X. Zhang X. Fu B. Xing J. Fu R. (2024). Hypoxia exacerbates the malignant transformation of gastric epithelial cells induced by long-term *H. pylori* infection. *Microbiol. Spectr.* 12:e0031124. 10.1128/spectrum.00311-24 38916312 PMC11302036

[B11] Hieken T. J. Chen J. Hoskin T. L. Walther-Antonio M. Johnson S. Ramaker S. (2016). The microbiome of aseptically collected human breast tissue in benign and malignant disease. *Sci. Rep.* 6:30751. 10.1038/srep30751 27485780 PMC4971513

[B12] Huang J. H. Wang J. Chai X. Q. Li Z. C. Jiang Y. H. Li J. (2022). The intratumoral bacterial metataxonomic signature of hepatocellular carcinoma. *Microbiol. Spectr.* 10:e0098322. 10.1128/spectrum.00983-22 36173308 PMC9602924

[B13] Lacombe A. Lee H. Zahed L. Choucair M. Muller J. M. Nelson S. F. (2006). Disruption of POF1B binding to nonmuscle actin filaments is associated with premature ovarian failure. *Am. J. Hum. Genet.* 79 113–119. 10.1086/505406 16773570 PMC1474115

[B14] Lee M. R. Tsai C. J. Liang S. K. Lin C. K. Huang Y. T. Hsueh P. R. (2015). Clinical characteristics of bacteraemia caused by Lactobacillus spp. and antimicrobial susceptibilities of the isolates at a medical centre in Taiwan, 2000-2014. *Int. J. Antimicrob. Agents* 46 439–445. 10.1016/j.ijantimicag.2015.06.017 26298673

[B15] Li Q. Wu W. Gong D. Shang R. Wang J. Yu H. (2021). Propionibacterium acnes overabundance in gastric cancer promotes M2 polarization of macrophages via a TLR4/PI3K/Akt signaling. *Gastric Cancer* 24 1242–1253. 10.1007/s10120-021-01202-8 34076786

[B16] Li Z. P. Liu J. X. Lu L. L. Wang L. L. Xu L. Guo Z. H. (2021). Overgrowth of Lactobacillus in gastric cancer. *World J. Gastrointestinal Oncol.* 13 1099–1108. 10.4251/wjgo.v13.i9.1099 34616515 PMC8465450

[B17] Ojeda D. Lakhal B. Fonseca D. J. Braham R. Landolsi H. Mateus H. E. (2011). Sequence analysis of the CDKN1B gene in patients with premature ovarian failure reveals a novel mutation potentially related to the phenotype. *Fertility Sterility* 95 2658–60.e1. 10.1016/j.fertnstert.2011.04.045 21575944

[B18] Ou S. Wang H. Tao Y. Luo K. Ye J. Ran S. (2022). *Fusobacterium nucleatum* and colorectal cancer: From phenomenon to mechanism. *Front. Cell. Infect. Microbiol.* 12:1020583. 10.3389/fcimb.2022.1020583 36523635 PMC9745098

[B19] Padovano V. Lucibello I. Alari V. Della Mina P. Crespi A. Ferrari I. (2011). The POF1B candidate gene for premature ovarian failure regulates epithelial polarity. *J. Cell Sci.* 124 3356–3368. 10.1242/jcs.088237 21940798

[B20] Pan K. F. Li W. Q. Zhang L. Liu W. D. Ma J. L. Zhang Y. (2024). Gastric cancer prevention by community eradication of *Helicobacter pylori*: A cluster-randomized controlled trial. *Nat. Med.* 30 3250–3260. 10.1038/s41591-024-03153-w 39079993

[B21] Parhi L. Alon-Maimon T. Sol A. Nejman D. Shhadeh A. Fainsod-Levi T. (2020). Breast cancer colonization by *Fusobacterium nucleatum* accelerates tumor growth and metastatic progression. *Nat. Commun.* 11:3259. 10.1038/s41467-020-16967-2 32591509 PMC7320135

[B22] Qiao H. Tan X. R. Li H. Li J. Y. Chen X. Z. Li Y. Q. (2022). Association of intratumoral microbiota with prognosis in patients with nasopharyngeal carcinoma from 2 hospitals in China. *JAMA Oncol.* 8 1301–1309. 10.1001/jamaoncol.2022.2810 35834269 PMC9284409

[B23] Smyth E. C. Nilsson M. Grabsch H. I. van Grieken N. C. Lordick F. (2020). Gastric cancer. *Lancet* 396 635–648. 10.1016/S0140-6736(20)31288-5 32861308

[B24] Stasiewicz M. Karpiński T. M. (2022). The oral microbiota and its role in carcinogenesis. *Semin. Cancer Biol.* 86 633–642. 10.1016/j.semcancer.2021.11.002 34743032

[B25] Strakova N. Korena K. Karpiskova R. (2021). *Klebsiella pneumoniae* producing bacterial toxin colibactin as a risk of colorectal cancer development - A systematic review. *Toxicon* 197 126–135. 10.1016/j.toxicon.2021.04.007 33901549

[B26] Tanaka Y. Tokubayashi Y. Kikuchi M. Fujii S. Kusaka T. Shibuya S. (2021). Gastric cancer with multiple lymph node enlargement at the time of the sarcoidosis diagnosis. *Intern. Med.* 60 3225–3229. 10.2169/internalmedicine.7218-21 33967139 PMC8580756

[B27] Urbaniak C. Cummins J. Brackstone M. Macklaim J. M. Gloor G. B. Baban C. K. (2014). Microbiota of human breast tissue. *Appl. Environ. Microbiol.* 80 3007–3014. 10.1128/AEM.00242-14 24610844 PMC4018903

[B28] Urbaniak C. Gloor G. B. Brackstone M. Scott L. Tangney M. Reid G. (2016). The microbiota of breast tissue and its association with breast cancer. *Appl. Environ. Microbiol.* 82 5039–5048. 10.1128/AEM.01235-16 27342554 PMC4968547

[B29] Wang G. He X. Wang Q. (2023). Intratumoral bacteria are an important “accomplice” in tumor development and metastasis. *Biochim. Biophys. Acta Rev. Cancer* 1878:188846. 10.1016/j.bbcan.2022.188846 36496095

[B30] Wang X. Zhao G. Shao S. Yao Y. (2024). *Helicobacter pylori* triggers inflammation and oncogenic transformation by perturbing the immune microenvironment. *Biochim. Biophys. Acta Rev. Cancer* 1879:189139. 10.1016/j.bbcan.2024.189139 38897421

[B31] Wang Z. Pei H. Liang H. Zhang Q. Wei L. Shi D. (2021). Construction and analysis of a circRNA-Mediated ceRNA Network in lung adenocarcinoma. *OncoTargets Therapy* 14 3659–3669. 10.2147/OTT.S305030 34135596 PMC8197624

[B32] Wong-Rolle A. Wei H. K. Zhao C. Jin C. (2021). Unexpected guests in the tumor microenvironment: Microbiome in cancer. *Protein Cell* 12 426–435. 10.1007/s13238-020-00813-8 33296049 PMC8106554

[B33] Zeng R. Gou H. Lau H. C. H. Yu J. (2024). Stomach microbiota in gastric cancer development and clinical implications. *Gut* 73 2062–2073. 10.1136/gutjnl-2024-332815 38886045 PMC11672014

[B34] Zhang M. Zhu J. Zhang P. Li L. Min M. Li T. (2023). Development and validation of cancer-associated fibroblasts-related gene landscape in prognosis and immune microenvironment of bladder cancer. *Front. Oncol.* 13:1174252. 10.3389/fonc.2023.1174252 37397364 PMC10309557

[B35] Zhu L. Huang Y. Li H. Shao S. (2022). *Helicobacter pylori* promotes gastric cancer progression through the tumor microenvironment. *Appl. Microbiol. Biotechnol.* 106 4375–4385. 10.1007/s00253-022-12011-z 35723694

